# Predictive value of gut microbiota in long-term blood pressure control: a cross-sectional study

**DOI:** 10.1186/s40001-022-00944-0

**Published:** 2023-03-12

**Authors:** Guobin Kang, Hongtao He, Huawei Miao, Tiejun Zhang, Zongde Meng, Xia Li

**Affiliations:** 1 Department of Cardiology, Hebei Province Hospital of Chinese Medicine, No. 389 of Zhongshan East Street, Chang’an District, Shijiazhuang, 050000 Hebei China; 2 Department of Internal Medicine, Hebei Province Hospital of Chinese Medicine, Shijiazhuang, 050000 Hebei China

**Keywords:** Hypertension, Intestinal microbiota, Diversity, Balance, Stool, Prediction, Blood pressure control

## Abstract

**Objectives:**

To investigate the prediction of long-term blood pressure control using the intestinal flora of patients with hypertension.

**Methods:**

A total of 125 patients with primary grade-2 hypertension who attended the cardiovascular department of Hebei Province Hospital of Chinese Medicine between April 2021 and April 2022 were enrolled; these included 65 patients with substandard long-term blood pressure control (the uncontrolled group) and 60 patients with standard long-term blood pressure control (the controlled group). General clinical data and data on morning stools and diet were recorded for all the enrolled patients. The 16 s rDNA sequencing of faecal intestinal flora was also performed to analyse the differences in intestinal flora between the two groups of patients and to investigate the relationship between blood pressure compliance and the presence of flora.

**Results:**

The intestinal flora of the two groups of patients differed in terms of the Firmicutes–Bacteroidetes ratio (F/B), α-diversity analysis (*Chao1, ACE and Shannon*) results and β-diversity analysis results. At the genus level, the number of *Streptococcus* and *Paraprevotella* in patients in the uncontrolled group was greater than that of the controlled group, and the level of *Akkermansia* and *Bifidobacterium* was lower than that in the controlled group. A logistic regression analysis of the difference factors found differences in ACE*,* F/B, *Streptococcus*, *Paraprevotella* and *Akkermansia* in the two groups; these differences remained after correcting for age, gender and body mass index. The receiver operating characteristic curves revealed the following: ACE (area under the curve [AUC] = 85.282), *Streptococcus* (AUC = 82.705), *Akkermansia* (AUC = 77.333), *Paraprevotella* (AUC = 66.154) and F/B (AUC = 60.436).

**Conclusions:**

There were significant differences in the intestinal flora of the patients in the controlled blood group compared with that of the uncontrolled group. Therefore, the ACE, genus levels of *Streptococcus* and *Akkermansia* could provide some prediction of late blood pressure compliance or non-compliance in patients with hypertension.

## Introduction

Hypertension is one of the most important and controllable risk factors for all-cause morbidity and mortality worldwide and is strongly associated with an increased risk of cardiovascular disease [1]. Although reductions in blood pressure can significantly reduce the occurrence of a wide range of acute events, long-term blood pressure control is required to reduce the global burden of disease and mortality [2]. Long-term substandard blood pressure control [3] or unstable control [4] can damage vital organs, such as the heart, brain, and kidneys, and it can lead to serious adverse events. The number of people with hypertension in China has reached 244.5 million [5]. However, the treatment rate of hypertension is less than 30%, and the average rate of achieving the standard is only 5.7% [6]. Therefore, the incidence of cardiovascular diseases caused by long-term substandard blood pressure control will remain high in China for many years.

It is well-known that hypertension is associated with genetics [7]. However, the human genome includes not only the deoxyribonucleic acid (DNA) inherited from parents but also the various flora (formed by interactions with the external physical environment after birth) that stably and harmoniously live within the body, accounting for up to 90% or more genome. Together, inherited parental DNA and intestinal flora form the human genome [8].

The abundance and number of intestinal flora vary according to human diseases, such as obesity, type-2 diabetes, non-alcoholic liver disease, malnutrition and hypertension [9]. Studies have shown that dietary modification can reduce the prevalence of hypertension in the population [10], and the absorption and metabolism of food are inevitably affected by intestinal flora and its metabolites. Current research confirms that the composition of intestinal flora and its metabolites, such as short-chain fatty acids, lipopolysaccharides and oxidized trimethylamine, influence the progression of cardiovascular disease [11]. Compared with healthy subjects, patients with hypertension have lower intestinal flora diversity, fewer short-chain fatty acid-producing microflora and more Gram-negative bacteria (which are sources of lipopolysaccharides) [12]. Furthermore, some animal studies have indicated that short-chain fatty acids directly regulate blood pressure, and lipopolysaccharides have significant pro-inflammatory effects [13]. This suggests that intestinal flora plays an important role in blood pressure regulation.

Most clinical studies have focused on investigating the relationship between intestinal flora and its related metabolites on the occurrence [14], development [15], treatment [16] and complications [17] of hypertension, and animal studies have focused on elucidating the mechanisms by which intestinal flora intervene in blood pressure [18, 19]; however, research on whether long-term blood pressure control in patients with hypertension is related to intestinal flora has not been reported.

Given the current situation of the long-term survival of patients being seriously affected by whether blood pressure standards are met or not, this study aimed to analyse the intestinal flora of patients with hypertension with and without standard blood pressure control; the aims were to explore the method of predicting late blood pressure control by the intestinal flora of patients with hypertension and to provide a basis for the achievement of blood pressure standards in patients with diagnosed hypertension.

## Subjects and methods

### Subjects

The study included 125 patients with primary grade-2 hypertension who attended the cardiovascular ward or outpatient clinic of Hebei Province Hospital of Chinese Medicine between April 2021 and April 2022. The subjects were enrolled into an uncontrolled group (65 patients with substandard long-term blood pressure control) or a controlled group (60 patients with standard long-term pressure control) according to whether blood pressure control had been achieved in the last month. The diagnosis of primary hypertension and blood pressure attainment were made with reference to the criteria in the *2018 ESC/ESH Guidelines for the Management of Arterial Hypertension* [20]. All enrolled patients provided signed informed consent, and the study protocol was approved by the ethics committee of Hebei Province Hospital of Chinese Medicine (acceptance number: 2020-KY-010–01).

#### Inclusion criteria

① Patients diagnosed with grade-2 simple hypertension (160–179 mmHg for systolic blood pressure and/or 100–109 mmHg for diastolic blood pressure) who were aged 18–80 years (including those aged 18 and 80 years). The highest blood pressure from a consultation or previous diagnosis was used to determine whether the enrolled patient met the diagnostic criteria for grade-2 hypertension. ② No adjustment of antihypertensive drugs in the past month and taking 1 × amlodipine benazepril tablet (2.5–10 mg) (Baianxin, Yangzijiang Pharmaceutical Group) regularly each day. ③ No consumption of drugs that may affect intestinal flora (e.g., probiotics, antimicrobials, diet pills, and laxatives) during the previous 3 months. ④ Regular diet and lifestyle and regular bowel movements in the past month. ⑤ Weight change < 5 kg in the past 3 months. ⑥ Voluntary participation in the clinical trial and willingness to provide signed informed consent.

#### Exclusion criteria

① Secondary hypertension caused by renal disease, renal artery stenosis, primary aldosteronism, pheochromocytoma, sleep apnoea, etc. ② Patients with combined non-hypertensive diseases. ③ A history of gastrointestinal diseases and gastrointestinal surgical diseases. ④ Other special conditions that may affect intestinal flora. ⑤ Patients on diets, those with weight loss and those with irregular lifestyles and eating habits.

### Methods

#### General data collection

General data, such as age, gender, smoking status, body mass index (BMI), duration of hypertension, blood pressure level and duration of medication, were recorded for both groups of subjects. It has been shown that exercise [21], diet [22] and yoghurt intake [23] can affect intestinal flora, so we divided the enrolled patients into three categories according to their weekly aerobic exercise level (< 3 times, 3–5 times and > 5 times), three categories according to their diet (meat-based, vegetable-based, and meat- and vegetable-based) and two categories according to their weekly yoghurt intake (≤ 300 ml and > 300 ml).

#### Stool specimen collection

All subjects fasted for 8–10 h, and their stools were collected in the early morning of the following day. ① Specimen retention: morning faeces were collected with a sterile collection spoon from the middle section of the stool (> 5 ml) and stored in a sterilized stool collector. ② Specimen storage: the stools were placed in a low-temperature refrigerator at − 80 °C within 1 h after collection for long-term storage before testing.

#### Intestinal flora assay

**Fig. 1:**
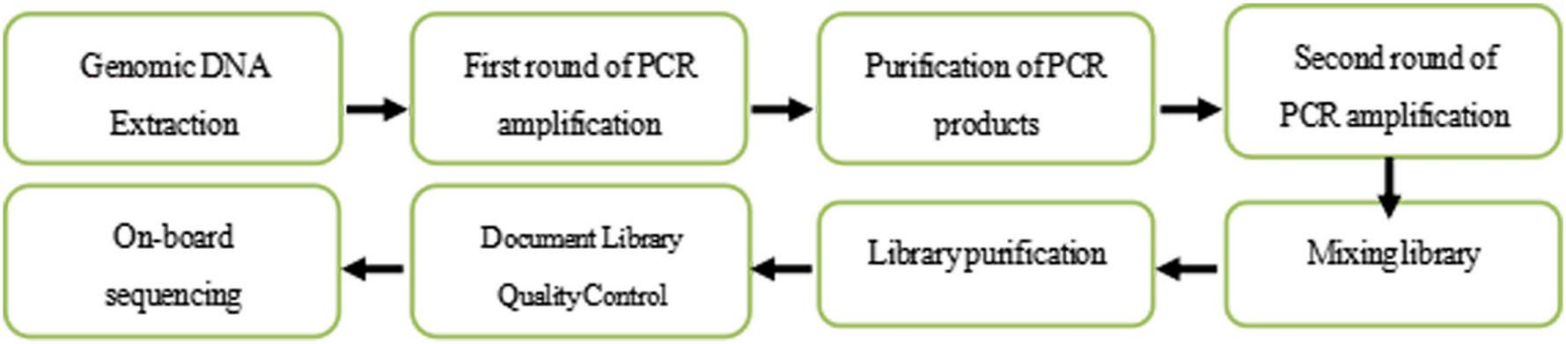
Flow chart of 16 s deoxyribonucleic acid assay for intestinal flora. *PCR* polymerase chain reaction, *DNA* deoxyribonucleic acid. In the raw data obtained from sequencing, there is a certain amount of interference data. To obtain high-quality sequencing data to improve the accuracy of the subsequent bioinformatics analysis, first, the original data needed to be spliced; then, they were quality controlled and filtered to obtain valid data

Deoxyribonucleic acid extraction was performed using a TIANGEN (Beijing, China) TIANamp stool DNA faecal genomic DNA extraction kit. This was followed by polymerase chain reaction amplification and purification, secondary amplification and purification, steps, such as library mixing and library processing, and finally, sequencing on the machine. The main steps are shown in Fig. 1.

### Statistical methods

All statistical analyses were performed using SPSS 17.0 statistical software. The measurement data that obeyed normal or approximately normal distributions were expressed as mean ± standard deviation ($\overline{x }$ ± *s*), and categorical data were expressed as numbers and percentages. An independent-samples *t* test was used for comparisons between the two groups when the variance was uniform, and a nonparametric test was used to compare the two groups when the variance was non-uniform. The aspects that initially screened out the differences between the two groups of patients were subjected to a binary logistic regression analysis to establish the factors affecting blood pressure compliance, and finally, the receiver operating characteristic (ROC) curve was plotted to test its predictive value. During the comparison, *P* < 0.05 was considered statistically different, and *P* < 0.01 was considered significantly different.

## Results

### General data of the patients in the two groups

There were no statistically significant differences between the two groups in terms of gender, age, smoking, BMI, duration of hypertension, duration of regular medication, exercise status, and diet (all *P* > 0.05). The systolic blood pressure (158.7 ± 7.95 vs. 126.6 ± 6.52 mmHg) and diastolic blood pressure (86.6 ± 6.53 vs. 74.6 ± 5.27 mmHg) were significantly higher in the uncontrolled group than in the controlled group, with a statistically significant difference (*P* < 0.01). The yoghurt intake of patients in the uncontrolled group was lower than that in the controlled group, and the difference was statistically significant (*P* < 0.05) (Table 1).

**Table 1:** Comparison of general information of patients in group A and group B

Factors	Uncontrolled group	Controlled group	*P* value
*n*	65	60	
Male (%)	30(46.2)	28(46.7)	> 0.05
Age (years)	68.5 ± 8.55	69.7 ± 8.07	> 0.05
Smoking (%)	28(43.1)	22(36.7)	> 0.05
BMI(kg/m^2^)	24.8 ± 2.45	24.8 ± 2.48	> 0.05
Duration of hypertension (months)	9.7 ± 4.72	10.0 ± 4.79	> 0.05
Duration of regular medication use (months)	2.42 ± 0.95	2.56 ± 1.00	> 0.05
Systolic blood pressure (mmHg)	158.7 ± 7.95	126.6 ± 6.52	< 0.01
Diastolic blood pressure (mmHg)	86.6 ± 6.53	74.6 ± 5.27	< 0.01
Exercise status (aerobic exercise) (%)			
< 3 times per week	9(13.8)	7(11.7)	> 0.05
3–5 times per week	22(33.8)	21(35.0)	> 0.05
Weekly > 5 times	34(52.3)	32(53.3)	> 0.05
Diet (%)			
Meat-based	4(6.2)	3(5.0)	> 0.05
Vegetarian-based	3(4.6)	3(5.0)	> 0.05
Meat and vegetables	58(89.2)	54(90.0)	> 0.05
Average intake of yogurt (%)			
≤ 300 ml per week	42(64.6)	25(42.7)	< 0.05
> 300 ml per week	23(35.4)	35(58.3)	

*BMI* body mass index

### Analysis of colony composition in the two groups of patients

#### Multilevel species diagram of the intestinal flora in the two groups of patients

**Fig. 2:**
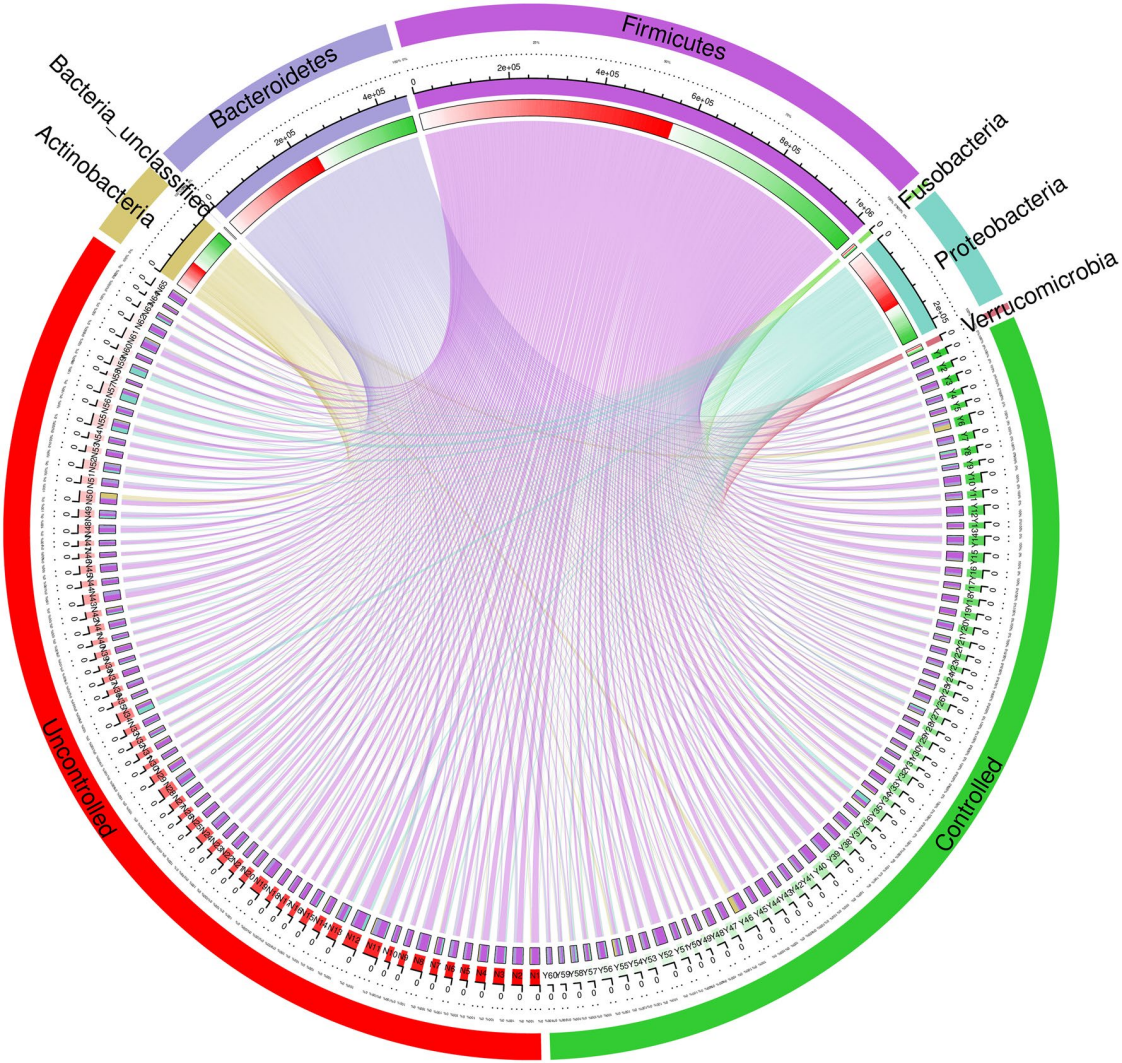
Multilevel species composition of faecal flora in the two groups of patients. This figure Shows the loop of the faecal flora phylum-level composition of the two groups of patients; the overall upper half is the classification of the phylum level of intestinal flora, and the lower half is the patient's intestinal flora specimen. The first circle (from the outside to the inside) represents the uncontrolled group, controlled group and the names of the phylum-level flora classification: Firmicutes, Bacteroidetes, Proteobacteria, Actinobacteria, Verrucomicrobia, Fusobacteria and Bacteria (unclassified). The second circle represents the number of each specimen in the two groups and the number of the phylum level of the flora. The third circle represents the percentage of phylum level in the two groups of specimens and the total percentage of the two groups. The linked line in the innermost circle shows the specific distribution of the phylum within the group

In the two groups, the highest to lowest proportions of intestinal flora of different phyla were Firmicutes, Bacteroidetes, Proteobacteria, Actinobacteria, Verrucomicrobia, Fusobacteria and Bacteria (see Fig. 2).

#### Comparison of the intestinal flora of the two groups of patients at the phylum level

**Fig. 3:**
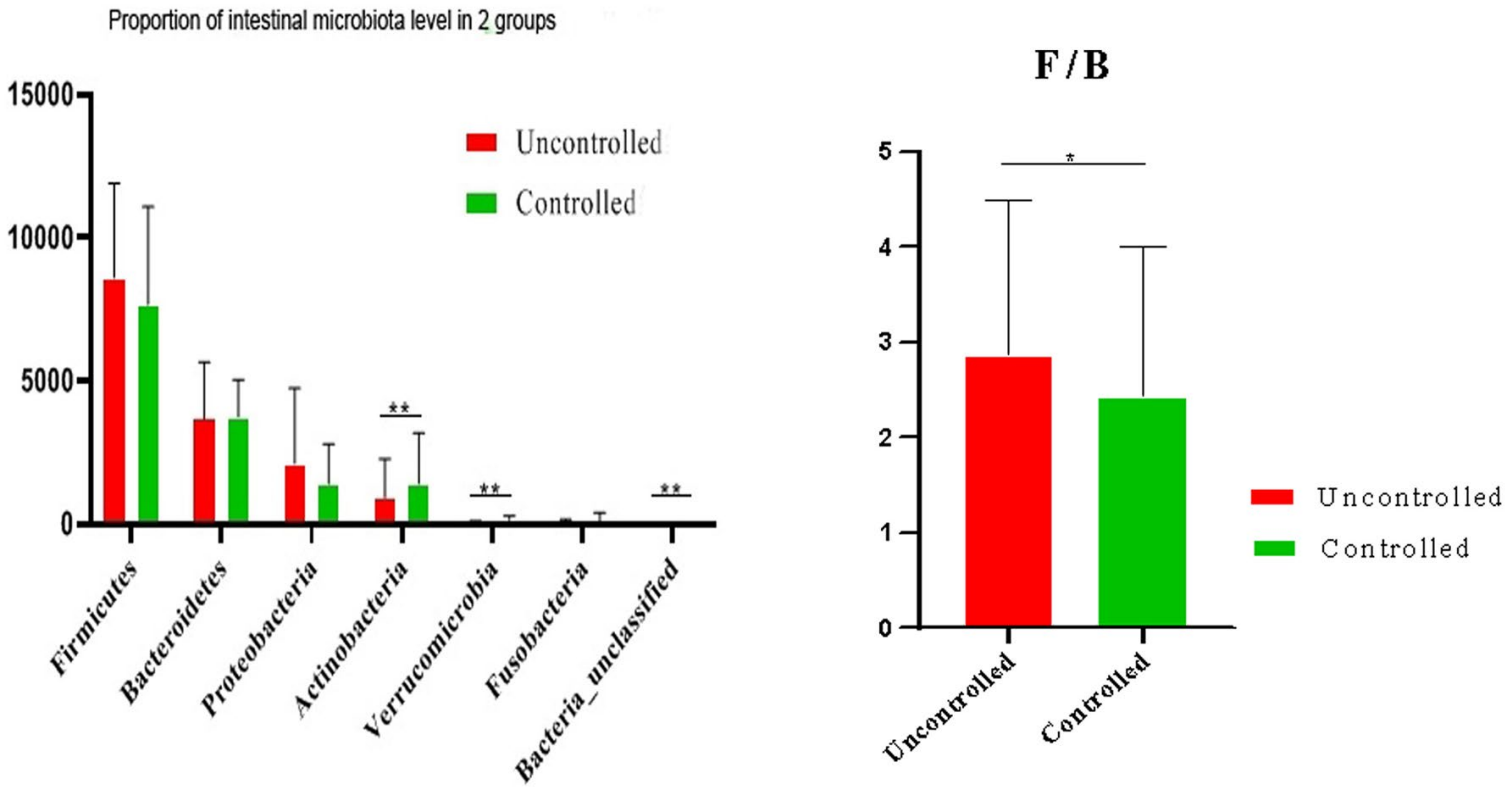
Comparison of faecal flora composition at the phylum level in the two groups of patients. F/B: Firmicutes–Bacteroidetes ratio, which is an important indicator of intestinal flora balance. The higher the F/B value, the worse the balance of the flora and the more serious the disorder of the flora [37]: Firmicutes, Bacteroidetes, Proteobacteria, Actinobacteria, Fusobacteria, Verrucomicrobia, Bacteria (unclassified). ^*^P < 0.05, ^**^ P < 0.01

There was no difference in intestinal flora between the uncontrolled and controlled groups of patients in terms of Firmicutes (8607.09 ± 3282.96 vs. 7669.87 ± 3408.64), Bacteroidetes (3666.12 ± 1989.33 vs. 3732.60 ± 1308.29), Proteobacteria (2101.82 ± 2646.66 vs. 1391.37 ± 1402.62) and Fusobacteria (58.09 ± 114.92 vs. 91.22 ± 304.10). There were differences in terms of Actinobacteria (901.42 ± 1368.94 vs. 1404.02 ± 1759.22), Verrucomicrobia (51.98 ± 58.65 vs. 140.82 ± 130.75), Bacteria (unclassified) (0.05 ± 0.28 vs. 9.02 ± 19.65) and the Firmicutes–Bacteroidetes ratio (F/B) (2.86 ± 1.64 vs. 2.42 ± 1.60) (see Fig. 3).

### Alpha diversity analysis

**Fig. 4:**
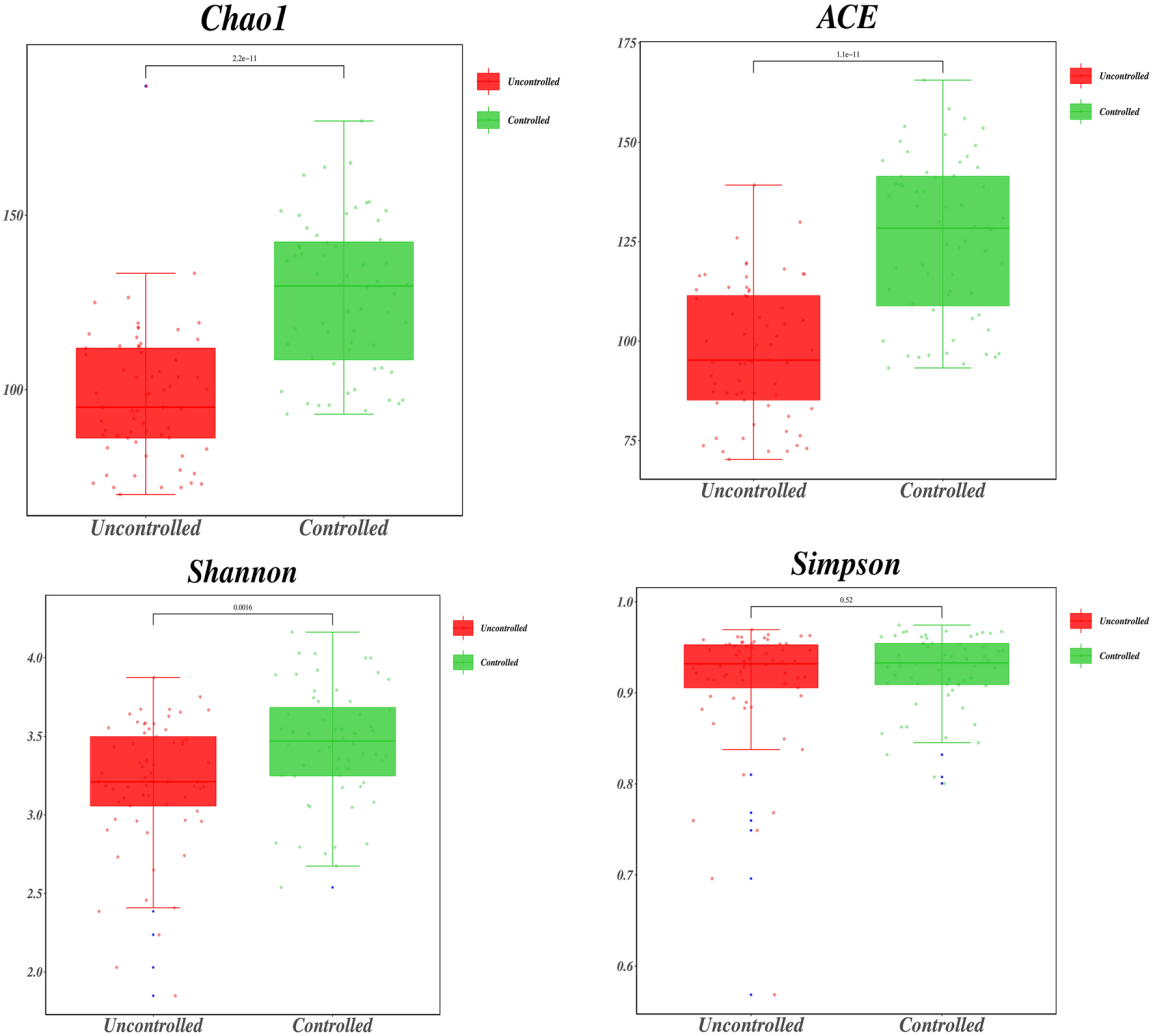
Comparison of intestinal flora abundance and diversity index in the two groups of patients. The Chao1 and ACE indices are mainly concerned with the species richness information of the samples and estimate the number of species contained in a colony, while the Shannon and Simpson indices mainly comprehensively reflect the diversity and evenness of species, i.e., high ACE and Chao1 indices indicate a high number of species in the samples, and high Shannon and Simpson indices indicate high species abundance and evenness

There was a significant difference between the two groups of patients in terms of *Chao1*, *ACE* and *Shannon* α diversity indices (*P* < 0.01), and there was no difference in terms of *Simpson* (*P* > 0.05) (see Fig. 4).

### Beta diversity analysis

**Fig. 5:**
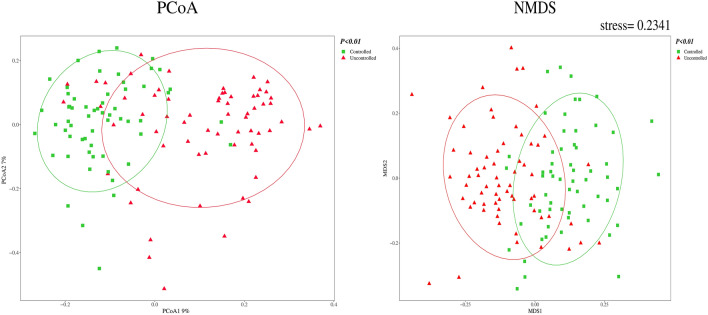
Comparison of the β diversity of the intestinal flora in the two groups of patients. *PCoA* principal coordinate analysis. During PCoA sorting, other distance/non-similarity matrices can be selected, and thus, the interrelationships between objects can be represented in two-dimensional coordinates. *NMDS* non-metric multidimensional scale analysis. This differs in that NMDS is no longer a characteristic root sorting technique and no longer aims to sort the bearings to load more variance; therefore, NMDS-sorted graphs can be arbitrarily rotated, centred and inverted. With the same number of axes, NMDS tends to obtain relationships between objects with less distortion than PCoA. The results of an NMDS analysis are measured by stress, which is generally considered to be represented by a two-dimensional point plot of NMDS when stress < 0.2 and its graph has some interpretative significance. When stress < 0.1, it can be considered a good ranking; when stress < 0.05, it is well-represented

A principal coordinate analysis (PCoA) and a non-metric multidimensional scale (NMDS) analysis were performed using the Bray–Curtis method, and the results showed a significant difference in intestinal flora between the two groups of patients (both *P* < 0.01), as shown in Fig. 5.

### Analysis of the differences in the genus levels of intestinal flora between the two groups of patients

**Fig. 6:**
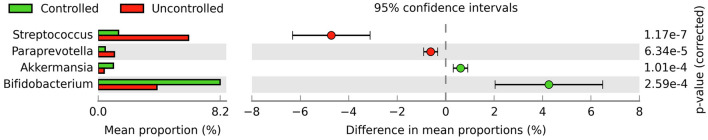
Differential analysis of the genus levels of intestinal flora in the two groups of patients. Each colour represents a group of samples. The bar on the left indicates the flora with significantly different abundance in the two groups of samples and the average relative abundance in the two groups, respectively. The graph on the right indicates the difference in relative abundance, separated by a dashed line in the middle; the left side of the dashed line indicates flora with higher relative abundance in one group, while the right side of the dashed line indicates flora with higher relative abundance in the other group; hence, each side of the dashed line is in a different colour. *Streptococcus*, *Paraprevotella*, *Akkermansia* and *Bifidobacterium*

There were differences in the genus levels of the intestinal flora of the patients in the uncontrolled and controlled groups. Considering the actual situation of the flora, there was a significant difference in terms of *Streptococcus* (891.71 ± 953.61 vs. 193.65 ± 214.90), *Paraprevotella* (148.11 ± 131.03 vs*.* 66.38 ± 45.69), *Akkermansia* (51.98 ± 58.65 vs*.* 140.82 ± 130.75) and *Bifidobacterium* (612.37 ± 607.45 vs*.* 1257.25 ± 1720.87) (*P* < 0.01) (see Fig. 6).

### Binary logistic regression analysis in terms of differences in intestinal flora between the two groups of patients

After the multifactorial regression analysis in the two groups of patients, the intestinal flora F/B [odds ratio (OR): 0.559, 95% confidence interval (CI) 0.336–0.930], *Streptococcus* (OR: 0.994, 95% CI 0.990–0.998) and *Paraprevotella* (OR: 0.978, 95% CI 0.964–0.993) were negatively associated with blood pressure attainment, and ACE (OR: 1.273, 95% CI 1.042–1.556) and *Akkermansia* (OR: 1.022, 95% CI 1.003–1.043) were positively correlated; this correlation persisted after correction for age, sex and BMI: F/B (OR: 0.548, 95% CI 0.327–0.919), *Streptococcus* (OR: 0.994, 95% CI 0.990–0.998), *Paraprevotella* (OR: 0.978, 95% CI 0.963–0.992), ACE (OR: 1.305, 95% CI 1.053–1.618) and *Akkermansia* (OR: 1.025, 95% CI 1.004–1.047) (see Table 2).

**Table 2:** Multi-factor binary logistic regression analysis of differential indicators in 2 groups of patients

Variables	OR	Multivariate		Variables	OR	Adjusted	
		95% CI	*P* value			95% CI	*P* value
				Gender	0.481	0.060–3.834	0.490
				Age	0.951	0.852–1.062	0.376
				BMI	0.920	0.626–1.351	0.671
F/B	0.559	0.336–0.930	0.025^*^	F/B	0.548	0.327–0.919	0.023^*^
*chao1*	0.904	0.768–1.064	0.226	*chao1*	0.895	0.761–1.052	0.179
*ACE*	1.273	1.042–1.556	0.018^*^	*ACE*	1.305	1.053–1.618	0.015^*^
*Shannon*	0.134	0.008–2.137	0.155	*Shannon*	0.074	0.003–1.698	0.103
*Akkermansia*	1.022	1.003–1.043	0.027^*^	*Akkermansia*	1.025	1.004–1.047	0.021^*^
*Streptococcus*	0.994	0.990–0.998	0.004^**^	*Streptococcus*	0.994	0.990–0.998	0.004^**^
*Paraprevotella*	0.978	0.964–0.993	0.004^**^	*Paraprevotella*	0.978	0.963–0.992	0.003^**^
*Bifidobacterium*	1.000	1.000–1.003	0.094	*Bifidobacterium*	1.001	1.000–1.003	0.111
Yogurt intake	1.798	0.283–11.414	0.534	Yogurt intake	1.874	0.257–13.685	0.536

*OR *odds ratio

^*^P < 0.05

^**^P < 0.01

### Value of intestinal flora for predicting blood pressure attainment

**Fig. 7:**
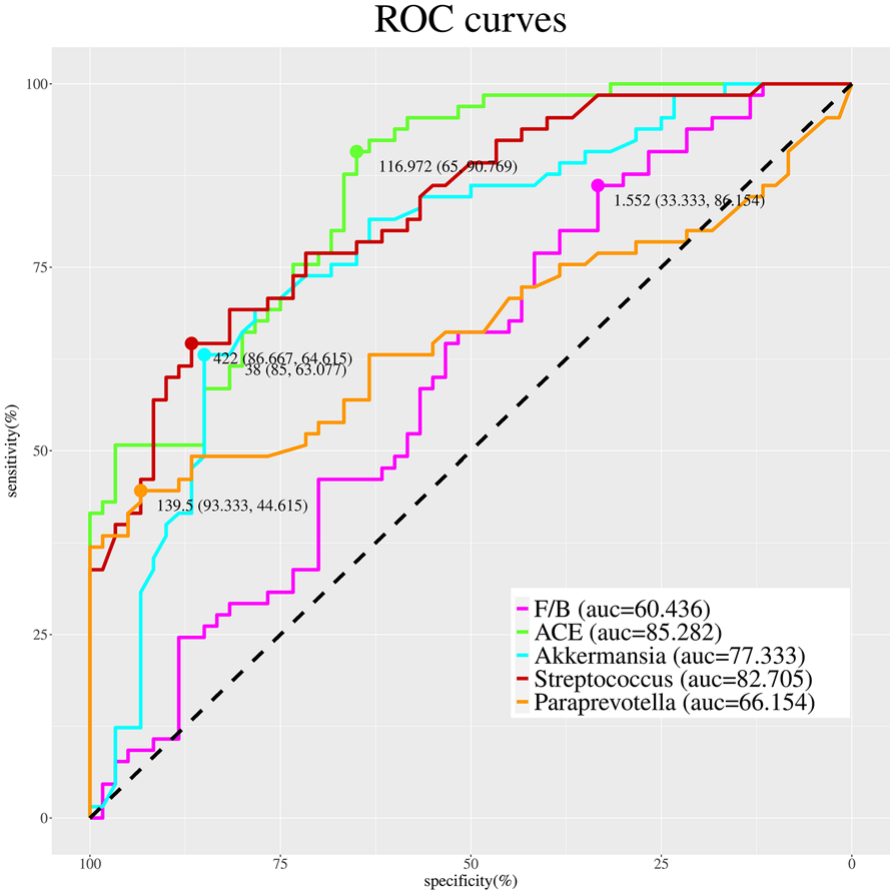
Receiver operating characteristic curves of factors of intestinal flora differences in the two groups of patients

The ROC curves were plotted for the variance factors derived from the multifactorial regression analysis, and the results showed that the florae were ranked from highest to lowest according to the predictive value: ACE (AUC = 85.282), *Streptococcus* (AUC = 82.705), *Akkermansia* (AUC = 77.333), *Paraprevotella* (AUC = 66.154) and F/B (AUC = 60.436) (see Fig. 7 and Table 3).

**Table 3:** ROC curve parameters

Variables	Area	Std. Error	Asymptotic Sig	95% CI	
				Lower bound	Upper bound
F/B	0.396	0.051	0.044	0.296	0.495
*ACE*	0.853	0.033	0.000	0.789	0.917
*Akkermansia*	0.773	0.043	0.000	0.690	0.857
*Streptococcus*	0.173	0.036	0.000	0.103	0.243
*Paraprevotella*	0.338	0.050	0.002	0.241	0.436

*ROC* receiver operating characteristic curve

## Discussion

A comparative analysis of the differential indicators between the two groups of patients and a multivariate regression analysis were performed to statistically identify indicators that may affect blood pressure attainment. The results revealed that intestinal flora F/B (OR: 0.559, 95% CI 0.336–0.930), the genus *Streptococcus* (OR: 0.994, 95% CI 0.990–0.998) and the genus *Paraprevotella* (OR: 0.978, 95% CI 0.964–0.993) were negatively associated with blood pressure attainment, and ACE (OR: 1.273, 95% CI 1.042–1.556) and the genus *Akkermansia* (OR: 1.022, 95% CI 1.003–1.043) were positively associated with blood pressure attainment. The differences persisted after correction for age, sex and BMI. The ROC curves for the genus level of differential bacteria were plotted to assess the predictive value of gut flora on blood pressure attainment, and the results revealed that ACE (AUC = 85.282), *Streptococcus* (AUC = 82.705) and *Akkermansia* (AUC = 77.333) had fair predictive specificity and sensitivity.

The general clinical data of the two groups of patients did not differ significantly in terms of age, BMI, exercise and diet, which may have interfered with the study outcome, but there were differences in yoghurt intake. Some studies have shown that probiotic supplementation can reduce blood pressure in patients with hypertension [24]. However, after a multifactorial analysis, the present study found no effect of yoghurt intake on blood pressure attainment, which may have been related to the small number of patients and the yoghurt intake classification. Patients differed in the comparison of phyla levels in terms of Actinobacteria, Verrucomicrobia, Bacteria (unclassified) and F/B indices. Only one F/B was finally included in the regression analysis, which is because during the analysis of this bacteriophage assay, under the detected phylum-level classification of Actinobacteria, other detected bacteria (e.g., *Senegalimassilia*, *Collinsella* and *Adlercreutzia*) were excluded from the genus-level comparison because of their low detection rates. Considering the actual clinical situation and to avoid the duplication of statistics, only the genus-level *Bifidobacterium* was included in the regression analysis without the phylum-level Actinobacteria; only one genus-level bacteria, *Akkermansia*, was analysed under the phylum level of Verrucomicrobia; this bacterium was different in the subsequent genus-level comparison, so a regression analysis was performed by genus level, and thus, it was not included. The phylum Bacteria (unclassified) was not included in the statistics, because it did not have any clinical application.

The F/B is an important indicator of intestinal flora balance: the larger the F/B value, the worse the flora balance and the more serious the flora disorder. Studies showed that the feeding of minocycline to pregnant and lactating rats resulted in an increased intestinal flora–F/B ratio and increased blood pressure in the offspring, accompanied by decreased levels of plasma acetate and butyric acid [25]. In another study, the exogenous supplementation of butyric acid or acetic acid in spontaneously hypertensive rats prevented an increase in blood pressure and an increase in the F/B ratio [26]. Furthermore, short-chain fatty acids are metabolites of intestinal flora, mainly butyric acid, acetic acid and propionic acid, with hypotensive, immunomodulatory and cardioprotective functions [27, 28]. These studies suggest that F/B is closely related to blood pressure, indicating that the intestinal flora of patients in the blood pressure attainment group in this study may provide more short-chain fatty acids to enhance the antihypertensive effect.

For the comparison of flora diversity, α diversity reflected the diversity, homogeneity and abundance of the distribution of the intestinal flora in the two groups of patients, and as previous studies in humans [29] and rats [30] had confirmed it to be correlated with blood pressure, it was included in the statistics. However, the β-diversity analysis only aimed to identify a significant difference between the two groups of flora and did not clearly propose the index of difference; it was used to describe the general difference in flora and to guide the subsequent analysis of the specific differences in the flora of the two groups of patients. The NMDS analysis was not included in the regression analysis, because the stress value was too high and might not have reflected the true situations of the two groups.

During the initial comparison of genus levels, a large number of differential bacteria were found, but from a practical point of view, the bacteria that were not identified at the genus level and those that were too small in number (the mean value of the genus level OUT in the two groups was < 10) were excluded. The bacteria that accounted for more than one-third of the blanks in both groups were also excluded. Finally, the four genus-level bacteria with differences were counted for the regression analysis. Comparing the differences in genus levels, *Streptococcus* and *Paraprevotella* were higher in the gut, and *Akkermansia* and *Bifidobacterium* were lower in the uncontrolled group compared with the controlled group. An increase in *Paraprevotella* and a decrease in *Akkermansia* have been observed in hypertensive rats during the progression from compensated cardiac hypertrophy to heart failure [31]. Chang et al. [32] found a lower abundance of *Bifidobacterium* in the intestinal flora of women with pre-eclampsia. Jin et al. [33] discovered that *Akkermansia*, propionic acid or butyric acid significantly reduced symptoms in rats with pre-eclampsia. Zhang et al. [34] found higher pharyngeal *Streptococcus* levels in patients with pulmonary hypertension compared with those of healthy subjects. Liu et al. [35] reported fewer genera of short-chain fatty acid-producing bacteria and more genera of *Streptococcus* associated with inflammation in the intestinal flora of patients with primary aldosteronism compared with the flora of a healthy group. It has also been reported that *Paraprevotella* is involved in the pathogenesis of hypertension in salt-sensitive rats [36]. The results of all these studies support those of the present research in one way or another.

The regression statistics of the indicators analysed for differences between the groups showed that intestinal flora F/B (OR: 0.559, 95% CI 0.336–0.930) and the genera levels of *Streptococcus* (OR: 0.994, 95% CI 0.990–0.998) and *Paraprevotella* (OR: 0.978, 95% CI 0.964–0.993) were negatively associated with blood pressure attainment, while ACE (OR: 1.273, 95% CI 1.042–1.556) and *Akkermansia* (OR: 1.022, 95% CI 1.003–1.043) were positively associated with blood pressure; this association persisted after correction for age, sex and BMI. The ROC curves for the predictive value of blood pressure compliance were plotted, with ACE (AUC = 85.282), *Streptococcus* (AUC = 82.705) and *Akkermansia* (AUC = 77.333) having the highest predictive values, providing some basis for later blood pressure compliance in patients attending clinics.

## Limitations

This study has some limitations. First, it was a single-centre cross-sectional study in which most of the participating population were patients living in the local neighbourhood. Therefore, a multicenter study is needed to expand the representativeness of the research. Second, although the sample size of this study was small, our research found a statistically significant correlation between intestinal flora and the anti-hypertensive effect of medication for grade-2 hypertension. Finally, only intestinal flora was analysed in this study, and subsequent analyses of blood and urine specimens from patients are required to reveal the mechanisms by which intestinal flora affects blood pressure. We plan to conduct a large-population multicenter study in the future to improve the credibility and extrapolation of our findings.

## Conclusions

There were significant differences in the intestinal flora of patients enrolled in the controlled blood pressure group compared with those in the uncontrolled group. The ACE genus levels of *Streptococcus* and *Akkermansia* could provide some prediction of late blood pressure compliance or non-compliance in patients with hypertension.

## Data Availability

All data generated or analysed during this study are included in this article.
